# Molecular docking and nucleotide sequencing of successive expressed recombinant fungal peroxidase gene in *E.coli*

**DOI:** 10.1186/s43141-022-00377-6

**Published:** 2022-07-01

**Authors:** Mohamed Khedr, Kamal M. A. Khalil, Hoda A. Kabary, Ahmed A. Hamed, Mona Shaban E. M. Badawy, Mohammed Abu-Elghait

**Affiliations:** 1 Department of Botany and Microbiology, Faculty of Science, Al-Azhar University, 11884 Nasr City, Cairo, Egypt; 2 Genetic Engineering and Biotechnology Division, Genetics and Cytology Department, National Research Centre, 33 El-Buhouth Street, Dokki, Cairo, 12622 Egypt; 3 Agricultural Microbiology Department, National Research Centre, 33 El-Buhouth Street, Dokki, Cairo, 12622 Egypt; 4 Microbial Chemistry Department, National Research Centre, 33 El-Buhouth Street, Dokki, Cairo, 12622 Egypt; 5 Department of Microbiology and Immunology, Faculty of Pharmacy (Girls), Al-Azhar University, Cairo, Egypt

**Keywords:** *Aspergillus niger*, Peroxidase PER-K, Recombination, PER-K nucleotide sequencing

## Abstract

**Background:**

Fungal peroxidases are oxidoreductases that utilize hydrogen peroxide to catalyze lignin biodegradation.

**Results:**

PER-K (peroxidase synthesis codon gene) was transformed from *Aspergillus niger* strain AN512 deposited in the National Center for Biotechnology Information with the accession number OK323140 to *Escherichia coli* strain (BL21-T7 with YEp356R recombinant plasmid) via calcium chloride heat-shock method. The impact of four parameters (CaCl_2_ concentrations, centrifugation time, shaking speed, growth intensity) on the efficacy of the transformation process was evaluated. Furthermore, peroxidase production after optimization was assessed both qualitatively and quantitatively, as well as SDS-PAGE analysis. The optimum conditions for a successful transformation process were as follows: CaCl_2_ concentrations (50 mM), centrifugation time (20 min), shaking speed (200 rpm), and growth optical density (0.45). PCR and gel electrophoresis detect DNA bands with lengths 175, 179, and 211 bps corresponding to UA3, AmpR, and PER-K genes respectively besides partially sequencing the PER-K gene. Pyrogallol/hydrogen peroxide assay confirmed peroxidase production, and the activity of the enzyme was determined to be 3924 U/L. SDS-PAGE analysis also confirms peroxidase production illustrated by the appearance of a single peroxidase protein band after staining with Coomassie blue R-250.

**Conclusion:**

A successful peroxidase-gene (PER-K) transformation from fungi to bacteria was performed correctly. The enzyme activity was screened, and partial sequencing of PER-K gene was analyzed successively. The protein 3D structure was generated via in silico homology modeling, and determination of binding sites and biological annotations of the constructed protein were carried out via COACH and COFACTOR based on the I-TASSER structure prediction.

**Supplementary Information:**

The online version contains supplementary material available at 10.1186/s43141-022-00377-6.

## Key points

- The optimization of *PER-K* fungal gene transformation in *Escherichia coli* was performed.
- The isoelectric point value of peroxidase is 6.98 with higher thermal stability.
- The 3D structures of the recombinant gene revealed high-quality model.

## Background

Peroxidases are a group of oxidative enzymes produced by living microbes either intra- or extracellular in a response to pollutant existence such as dyes and aromatic hydrocarbons in the same surrounding environments [1]. These compounds are oxidized through a free radical mechanism to render the products less toxic to the living cell either through loss of biological activity or reduction in the bioavailability or adsorption. Peroxidase enzymes catalyze oxidative reactions via hydrogen peroxide utilization and protect the microorganisms from oxidation by free radicals [2]. These enzymes have been involved in different biotechnological purposes such as cosmetics, pulping, paper manufacturing, and wastewater treatment [3]. Fungal peroxidase plays an important role in lignin biodegradation and other environmental applications, such as biobleaching, biopulping, and bioremediation of soil. Therefore, ligninolytic enzymes of fungal origin have been extensively studied [4, 5]. White-rot fungi *Phanerochaete chrysosporium* is the most resplendent producing strain for lignin-degrading enzymes. Both lignin peroxidase *lipA* and manganese peroxidase *mnp* genes in *Aspergillus niger* have been extensively isolated in several studies [6, 7]. The wide application of peroxidase production for industrial potentials is limited due to low yield and expensive production cost. The increased demands for peroxidase production shed the light to discover new microorganisms capable of efficient peroxidase production. For feasible and efficient industrial applications, large-scale production for these enzymes is needed. Peroxidases availability in the market including *Bjerkandera adusta* peroxidase, horseradish peroxidase [HRP], and streptavidin peroxidase from *Streptomyces avidinii* probably could not meet the increasing industrial demand for peroxidase [1]. Recombinant peroxidase enzyme technique is one of the best ways to achieve this purpose [8]. This technique is the calcium chloride heat-shock transformation process which enhances bacterial cells to uptake DNA from the surrounding environment. The calcium ions role is predicted to be a cation bridge in the cell suspension between the phosphate backbone of DNA and negative charges on phosphorylated lipid A in lipopolysaccharide (LPS) [9, 10]. The ice-cold CaCl2 solution activates the binding of DNA to the surface of the cell, which then enters the cell by a short period of heat shock [11]. Selection markers such as drug resistance are usually used to identify the successful transformation of the interested gene [12]. This technique is usually used to transform cells with plasmids for cloning, recombinant protein expression, and long-term storage of the plasmids [13]. Therefore, the present study aimed to transform the competent *E. coli* BL21-T7 with recombinant plasmid carrying *A. niger* peroxidase enzyme coding gene, to confirm the peroxidase enzyme production by the new recombinant *E. coli*, and to determine the culture conditions that support optimum peroxidase production recombinant strain. In addition, peroxidase production from transformed *E. coli* was estimated qualitatively and quantitatively as a new potential applicable source for extracellular peroxidase production.

## Material and methods

### Microbial strains and plasmid isolation

Two strains, *E. coli* BL21 — T7 and *Aspergillus niger* AN512, were cultivated from glycerol stock in nutrient agar (NA) (Oxoid, USA) and potato dextrose agar (PDA) (Oxoid, USA) for 24 h and 4 days for bacterial and fungal strains, respectively. Shuttle plasmid YEp356R (1.0μg/μl) ATCC 37737 was *previously isolated using EasyPure* Plasmid MiniPrep Kit, from *E. coli* Dh5α-k12. Selective step is carried out on MacConkey agar (Oxoid, USA) supplemented with 50 μg/ml ampicillin (Fig. S1) [14, 15]. All bacterial, fungal, and plasmid samples were obtained from Applied Microbial Genetics Department, Genetic Engineering and Biotechnology Division, NRC, Giza, Egypt.

### Preparation of competent cells

Different concentrations of CaCl_2_, 0, 25, 50, 75, 100, 125, 150, and 200 mM were prepared, and improved buffer was prepared as follows: add 100 ml of glycerin into 50 mM CaCl_2_ solution, and then, complete the final volume to 1000 ml with ddH_2_O. *E. coli* BL21-T7 cells were picked from NA and grown on Luria-Bertani (LB) broth medium (Oxoid, USA) under vigorous incubation shaking condition (200 rpm) for 10 to 16 h until it reaches optical density (OD_600_) of 0.2 to 0.45. The resulted cell pellet was harvested by centrifugation undercooling (−4 °C) at 10,000 rpm for 5 min and washed three times by CaCl_2_ (50 mmol) supplemented with 17% glycerin. Finally, the cells were suspended in CaCl_2_ buffer and distributed in 1.5 ml Eppendorf [14, 15].

### Construction of recombinant plasmid

After plasmid isolation, it digested with endonuclease buffers. The desired gene PER-K was also isolated from fungal DNA after its extraction with the same restriction enzymes which give complementary ends in plasmid and gene for easily recombination step. Digestion process was composed of 500 μl of DNA with 55 μl from both endonuclease buffers (1 μg DNA restriction 0.5 μg), and this ratio is also used for plasmid digestion. Restriction endonucleases and digestion buffers were illustrated in Tables S1 and S2.

### Optimization of transformation conditions

Several steps were performed according to [16] for the determination of the efficiency of the transformation process. The different culture conditions, such as shaking speed (50, 70, 90, 120 150, and 200 rpm), growth turbidity using OD_600_ values (0–0.5), and centrifugation time (5, 10, 15, and 20 min), were tested to prepare *E. coli* competent cells and assessed by calculating the transformation efficiency. All optimization items were established in triplicates.

### Genomic detection of successful transformant

The recombinant plasmid was isolated and extracted using QuickClean II Plasmid Purification Kit 50 rxns. Then, the plasmids were screened for their purity and concentration through NanoDrop and agarose gel electrophoresis, the appropriate samples were prepared for PCR reaction as a template, and specific primers were used for amplification plasmid genes (Per-K, UA3, and ampR). The primers used were illustrated in Table 1.

**Table 1:** DNA-specific primers of the studied genes amplifications

Gene	Primers	Nucleotide sequence	GC%	Tm °C	Product size
Peroxidase	F-POX1	5-CCAAATCTCCTCTTCCTTA-3	42.1	56.8	211
	R-POX2	5-TGTCCTCTAACACTTCTCGCA-3	47.6	58	
URA	F-URA3	5-GCAAGGGCTCCCTAGCTACT-3	60	60	175
	R-URA3	5-AATGCGTCTCCCTTGTCATC-3	50	60.1	
AmpR	F-AmpR	5-GAGTATTCAACATTTCCG-3	52	61.6	179
	R-AmpR	5-CGGGGCGAAAACTCT-3	65	36.2	

PCR detection and amplification of desired gene Per-K as well as two plasmid gene markers were performed under different specific conditions according to each gene. For *Per-K*, the PCR conditions were denaturation step for the first cycle adjusted at 95 °C for 1 min, extension step for the final cycle was at 4 °C overnight, while the rest of other cycles of the reaction was denaturation cycles (2–34) at 95 °C for 30 s, annealing cycles (2-34) at 56 °C for 30 s, and extension (cycles 2–34) at 72 °C for 40 s (Table 1).

In case of *URA3*, PCR conditions of denaturation step for the first cycle are adjusted at 93 °C for 1 min, and extension step for the final cycle was at 4 °C overnight, and the rest of other cycles of the reaction was as follows: denaturation cycles (2–37) at 93 °C for 30 s, annealing cycles (2–37) at 59 °C for 30 s, and extension (cycles 2–37) at 73 °C for 40 s (Table 1).

Regarding *Amp*^R^, PCR conditions comprised of denaturation step for the first cycle adjusted at 93 °C for 1 min, and extension step for the final cycle was at 4 °C overnight, and the rest of other cycles of the reaction was as the follows: denaturation cycles (2–33) at 93 °C for 30 s, annealing cycles (2–33) at 55 °C for 30 s, and extension (cycles 2–33) at 74 °C for 40 s (Table 1).

### Isolation and cloning per-K gene

per-K gene is isolated from fungus through total genomic DNA extraction. Its purity was determined via NanoDrop and estimated at about 50 μg/ml. The extracted DNA was then digested with restriction digestion solution SalI digestion and HindIII (Table S2). On the other hand, plasmid was isolated from the *E. coli* host and its purity was determined by NanoDrop and estimated 1.00, and it also digested with the same restriction solutions that are mentioned before (Table S2). The recombination step is done in a 1.5 mL Eppendorf tube that is kept on the ice at −20 °C with different ratios of plasmid and digested DNA for the DNA library. The successful step was composed of 300 μl of digested DNA with 100 μl plasmid solution, and then, this mixture was mixed at very slow rotation speed with a thin, alcohol-sterilized glass rod in a clockwise direction for 15 s. After that, the mixture was kept in water bath 32 °C for 15 min; finally, it was mixed with sterile M9 medium with glucose and incubated without shaking for 4 h before transferring to LB broth with ampicillin for selecting our recombinant.

### Determination of peroxidase molecular weight by SDS-PAGE

The molecular weight of peroxidase protein was determined through SDS–PAGE in a Mini Protean III Electrophoresis Cell (Bio-Rad), with 12% resolving and 4% stacking gel. Proteins were stained using the Coomassie blue staining technique, and the molecular weight was estimated by comparison to molecular weight ladder (10 to 180 kDa).

### Detection of peroxidase activity

Peroxidase activity was evaluated using the method of Rayner and Boddy (1988). Briefly, a 2-day incubated culture at 30 °C was grown in nutrient agar plates, and the resulting colonies were immersed with 100 μl of 0.4% (v/v) hydrogen peroxide and 1% pyrogallol in water. Colonies that developed yellow-brown color was considered as positive for peroxidase production [17].

### Total protein concentration in culture filtrate

Culture filtrate total protein was determined and calculated using the Bradford method for the quantitative determination of peroxidase activity. The isolate was incubated in tryptone soy broth (TSB) medium for 5 days, and the culture supernatant was separated by centrifugation at 6000 rpm for 25 min. A total of 50 μl of the supernatant was transferred to a clean test tube, and the volume was brought to 1 ml with phosphate buffer (0.1 M, pH 6.5). Five milliliters of protein reagent solution “Coomassie brilliant blue G-250 in ethanol” was added. The final reading was detected spectrophotometrically at 595 nm after 2 min and before 1 h of the reagent addition. The amount of total protein detected was calculated in μg/mL according to the standard equation: *Y* = 9011.1x–157.98 where x = absorbance at 595 nm [18].

### Quantitative determination of peroxidase activity

Peroxidase activity was evaluated according to the rate of hydrogen peroxide oxidation of pyrogallol to purpurogallin as exhibited by modified method of Chance and Maehly. Reaction mixture of 350 μl was prepared, composed of 5% w/v pyrogallol dissolved in 100 mM potassium phosphate buffer (pH 6) and 25 μl of 5 days’ culture supernatant. The reaction mixture without culture supernatant was prepared as blank. The reaction was begun by the addition of 0.5% hydrogen peroxide (30%), and the readings were measured spectrophotometrically from the beginning with an interval of 30 s for 3 min at 420 nm [19, 20]. Peroxidase activity was calculated according to the equation U/ml: abs. (final)—abs. (beginning)/0.001.

### Analysis of physicochemical parameters of peroxidase enzyme

To compute the physicochemical parameters of the peroxidase protein, ExPASy’s ProtParam program was used. These properties can be derived from a protein sequence which includes parameters such as molecular weight (M.Wt), instability index (II), aliphatic index (AI), theoretical pI, and grand average of hydropathicity (GRAVY). The instability index provides an approximation of our protein’s stability, an instability index less than 40 is projected to be stable, and a score greater than 40 indicates that the protein may be unstable [21].

### Construction of the 3D enzymes structure by homology modeling

The amino acid sequences of the *Aspergillus niger* peroxidase enzymes were submitted to the SWISS-MODEL, and the 3D structure of the peroxidase enzymes was automatically generated by first transferring conserved atom coordinates provided by the desired template alignment [22]. Endo β-1,4-glucanase from *Bacillus licheniformis* was utilized as a template to perform the homology modeling of the fungal cellulase structure. The enzyme models were obtained as a PDB file, and the model was energy minimized via Gromos96 tools in the Swiss-PDB viewer [23].

### Identification of the enzymes catalytic residues

The active-site residues of the peroxidase enzyme were predicted using the I-TASSER web server (https://zhanggroup.org/I-TASSER/). I-TASSER web server detects catalytic residues in the primary structural alignment, which was then viewed in PyMOL. According to a previously reported approach, the probable active-site residues were superimposed on a template structure in this case [24]. COACH, a meta-server, was then used to predict the protein-ligand interaction site. To construct the final ligand binding site predictions, the predictions were merged with data from the COFACTOR, FINDSITE, and ConCavity analyses.

## Results

### Positive transformant selection

Out of 225 isolated colonies, about 14 single colonies were able to grow on M9 minimal medium supplemented with 1% glucose and incubated at 37 °C for 24 h with the lowest shaking (50 rpm), nutrient agar medium was also used to cultivate the promising 14 colonies as standard plates. The fourteen different transformant colonies were confirmed for successful cloning with the recombinant plasmid through cultivation on MacConkey agar supplemented with 30 μg/mL ampicillin. All colonies were grown well with pinky color. MacConkey agar is considered a good selective medium for our *E. coli* host strain as it was gram-negative lactose-fermenting bacteria besides the presence of ampicillin allows us to easily pick up grown cells with successful recombinant plasmid.

### Optimization of plasmid transformation

**Fig. 1:**
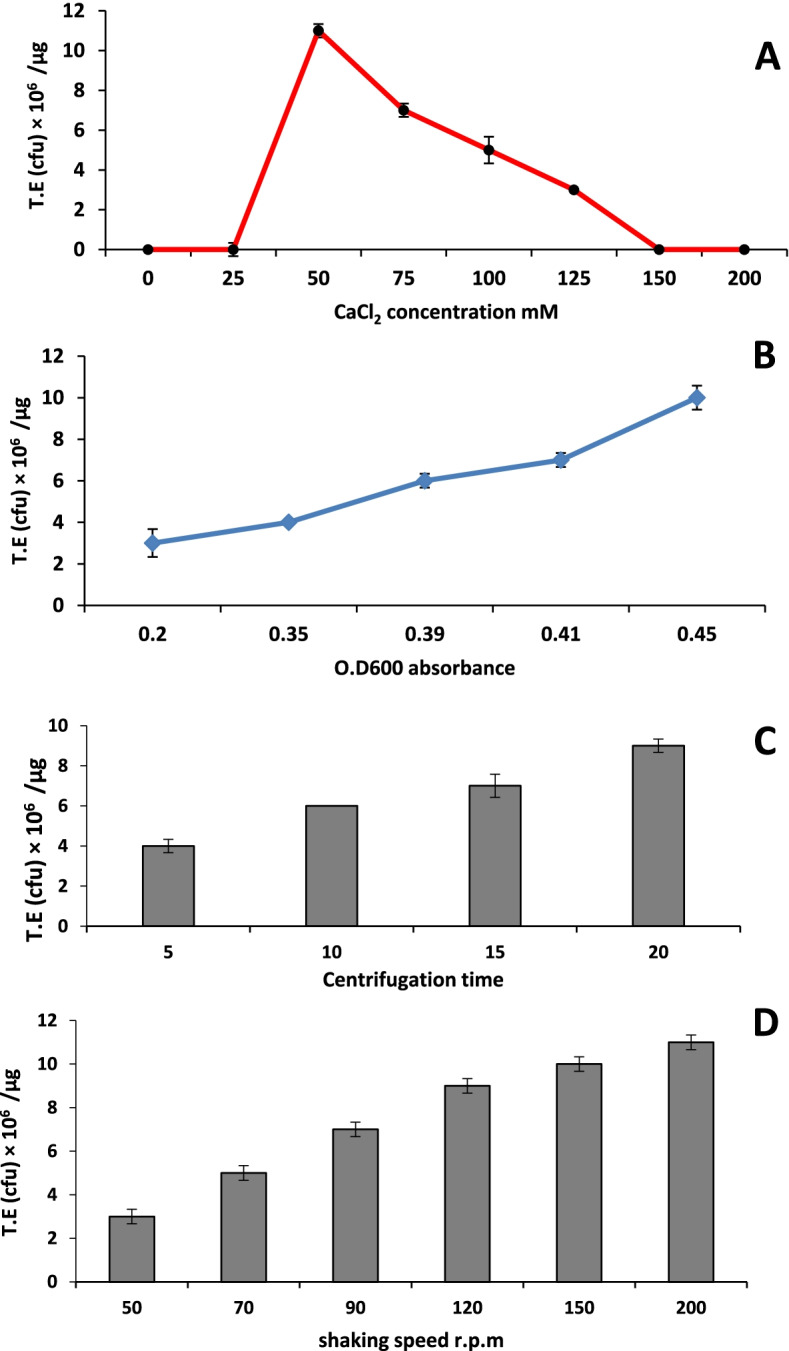
Optimization for plasmid transformation. **a** CaCl2 concentrations. **b** Different absorbance values. **c** Different centrifugation time. **d** Different shaking speeds. TE, transformation efficiency

Optimization for plasmid transformation was performed through CaCl_2_ adjustment, so different CaCl_2_ solution concentrations were used, and transformation efficiency increased at concentration 25 mM and gradually increased by increasing solution concentration and reached its maximum value at 50 mM of CaCl_2_ solution and then decreased gradually until 150 mM (Fig. 1a). In the present study, the highest transformation efficiency reached its maximum when the OD_600_ values were between 0.41 and 0.45 (Fig. 1b). The transformation efficiency of competent cells was also affected by centrifugation time where it was neglectable value at centrifugation less than 5 min and begun to increase after 5 min, and its efficiency reaches a maximum at 20 min and then decreased gradually until be neglectable again (Fig. 1c). Shaker’s speed during competent cells preparation is also considered a significant factor in the transformation efficiency. Transformation efficiency in our study was increased gradually from 50 rpm until it reached its maximum of 11 × 10^6^/μg at 200 rpm (Fig. 1d).

### PCR detection of the recombinant plasmid

Three genes (our insert per-K, UA3, and Amp^R^ genes) were used as target sequences for PCR amplification in order to detect our insert plasmid into host cells. Agarose gel electrophoresis and DNA sequences (Fig. S2) showed that Per-K, UA3, and Amp^R^ amplicons were 211, 175, and 179 bps, respectively. Per-K sequence scored through online BLAST, 100% query cover, with a total and maximum score of 390 with *Aspergillus officinalis* peroxidase 72-like (LOC109840690), mRNA (Figs. S3 and S4 Table 2).

**Table 2:** The highest six sequences that give high similarity with the peroxidase gene of our interest

Description	Scientific name	Max score	Total score	Query cover	Per. ident	Acc. len	Accession
Peroxidase 72-like (*Asparagus officinalis*)	*A. officinalis*	689	689	75%	100.00%	334	XP_020265010.1
Peroxidase 72-like (*Asparagus officinalis*)	*A. officinalis*	554	554	75%	79.17%	329	XP_020272526.1
Peroxidase (*Asparagus officinalis*)	*A. officinalis*	550	550	75%	78.57%	329	BAA94962.1
Peroxidase 72 (*Elaeis guineensis*)	*E. guineensis*	547	547	75%	79.04%	331	XP_010924103.1
Peroxidase 72 (*Elaeis guineensis*)	*E. guineensis*	541	541	75%	76.58%	330	XP_010936646.1
Peroxidase 72-like (*Phoenix dactylifera*)	*P. dactylifera*	541	541	75%	78.74%	331	XP_038987667.1

### PER-K gene isolation and cloning

The complete gene sequence was analyzed. The open reading frame (ORF) started from 141 to 1145 bps, while the total nucleotide sequence was 1330 bps (Fig. S5). The amino acid polypeptide resulted from ORF was 334 which gives protein with an expected molecular weight near to 33.4 KDa as analyzed through SnapGene Viewer software (version 4.1.3).

### Partial purification of peroxidase and molecular weight determination

**Fig. 2:**
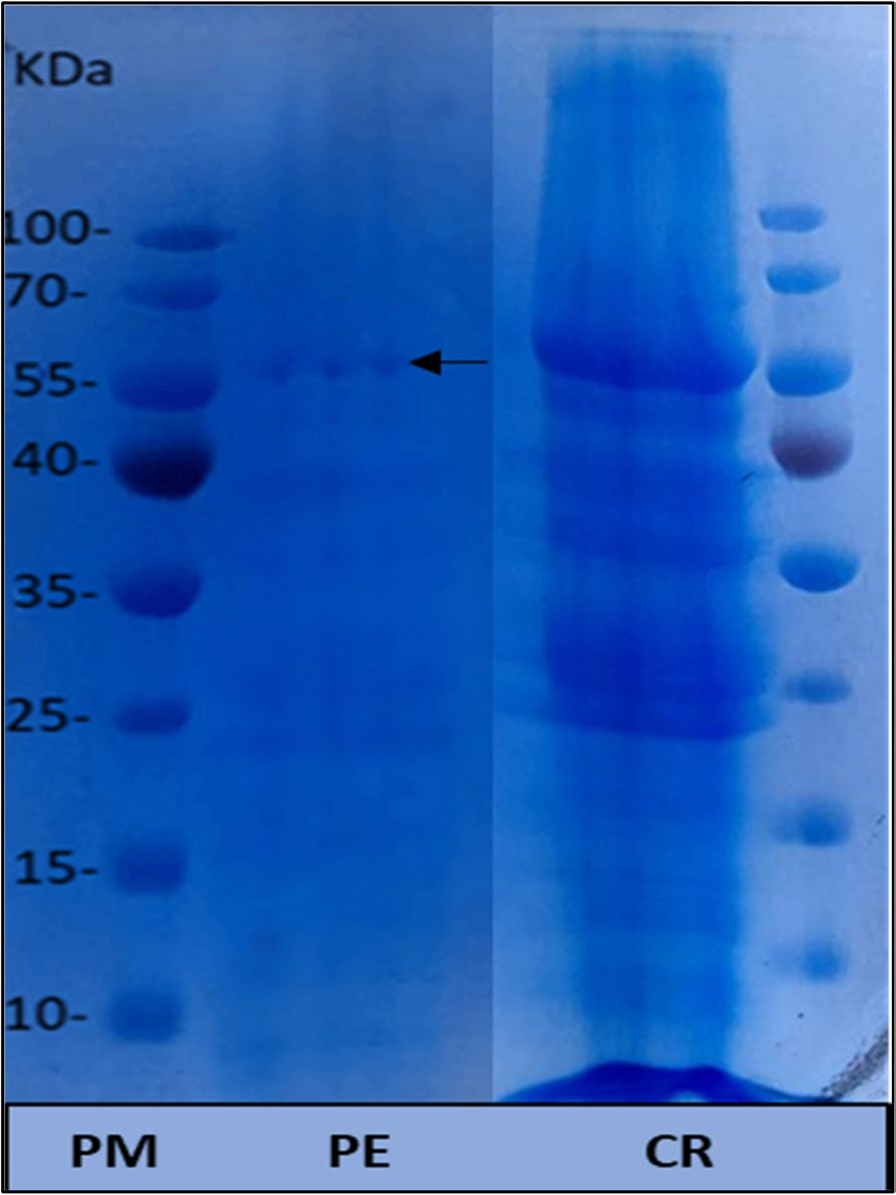
Molecular weight of recombinant peroxidase by electrophoretic analysis on 7.5% SDS-PAGE. PM, molecular weight marker proteins. PEP, purified peroxidase. CR, crude extract

The results showed that 5.9% of ammonium sulfate was given complete precipitation of the enzyme with maximum enzyme activity. After the precipitation of peroxidase with ammonium sulfate, the sediment was redissolved in citrate-phosphate buffer (pH 7.2) and dialyzed against the same buffer. The obtained purified bacterial peroxidase showed a single protein band (55 KDa) on SDS-PAGE after staining with Coomassie blue R-250 (Fig. 2).

### Peroxidase activity

*E. coli* cloned strain was detected as a positive for peroxidase enzyme production, illustrated by the appearance of yellow-brown color after immersing growth with 1% pyrogallol and 0.4% hydrogen peroxide. The quantitative evaluation of peroxidase production and activity was calculated by subtracting the final absorbance of the reaction mixture (0.456) from the initial absorbance (0.064) at 420 nm divided by 0.001. The obtained peroxidase enzymatic activity detected was 392.4 U/ml after 5 days of the incubation period.

### Physicochemical properties of peroxidase

The ProtParam tool was used to predict the physicochemical parameters of the peroxidase protein enzyme. The physical parameters showed that the enzyme’s molecular weight is 36,650.92 Da, and the peroxidase enzyme’s instability index is 48.26. *Aspergillus niger* AN512 peroxidase has a calculated isoelectric point pI value of6.98 and a higher aliphatic index (85.57), suggesting that it is a thermally stable protein. Meanwhile, the peroxidase enzyme’s negatively grand average of hydropathicity (GRAVY) values revealed its hydrophilicity (−0.156). The total number of negatively charged residues (Asp + Glu) is 37, while the total number of positively charged residues (Arg + Lys) is also 37 (Table 3).

**Table 3:** Summary of the ProtParam data for the *Aspergillus niger* AN512 peroxidase

Details	Peroxidase
Amino acid residue	334
Molecular weight	36650.92
Theoretical pI	6.98
Positively charged residue	37
Negatively charged residue	37
Total no. atoms	5144
Molecular formula	C_1626_H_2571_N_441_O_490_S_16_
Aliphatic index (%)	85.57
Instability index (%)	48.26
GRAVY	−0.156

### Modeling the 3D structures of Aspergillus niger AN512 peroxidase enzymes

**Fig. 3:**
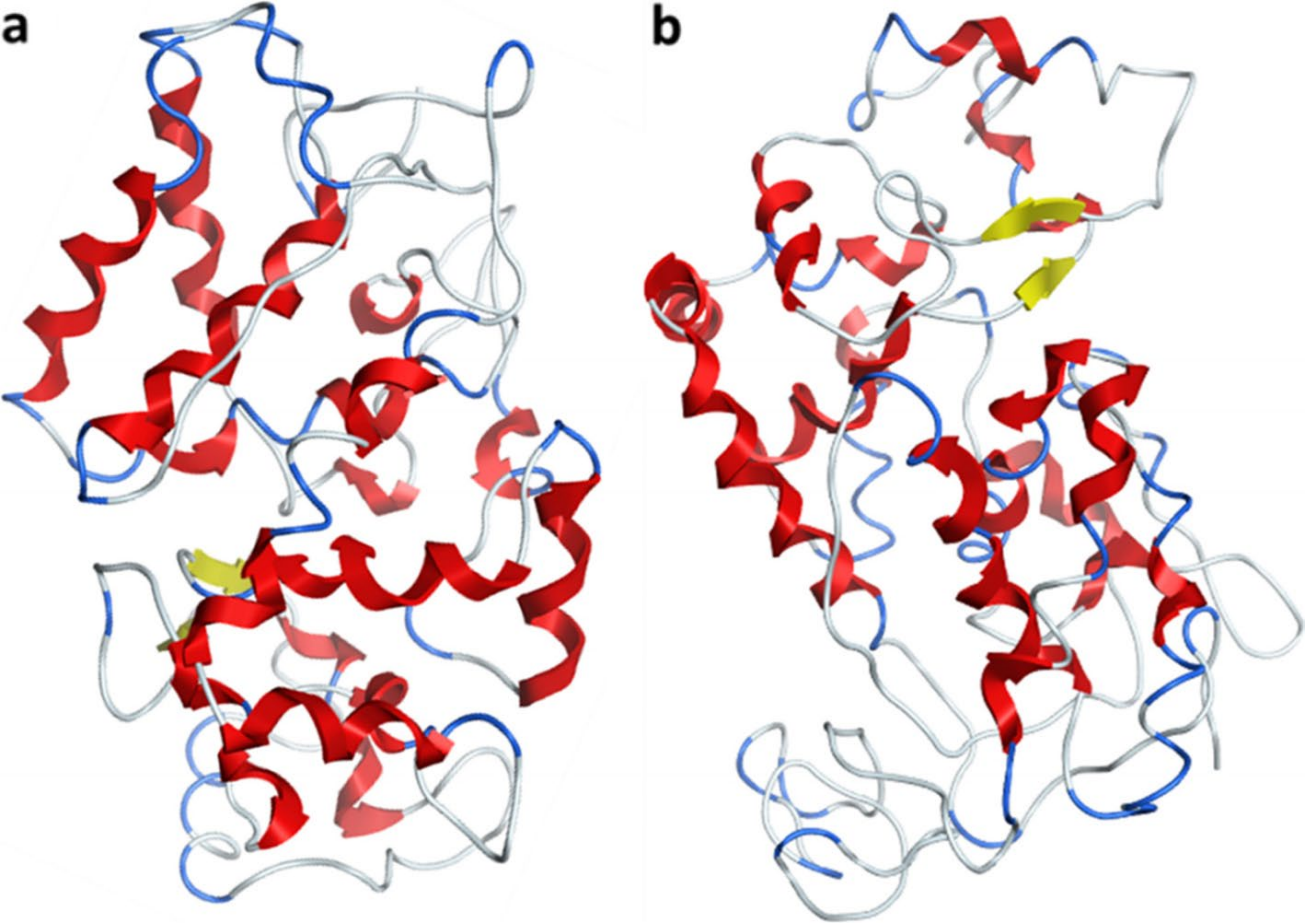
**a** The SWISS-MODEL generated 3D structures of *Aspergillus niger* AN512 peroxidase enzymes. **b** The predicted 3D structures by I-TASSER

The amino acid sequences of *Aspergillus niger* AN512 peroxidase were subjected to homology modeling via SWISS-MODELweb server to generate the 3D structures of the peroxidase enzyme. The cellulase enzyme was constructed using an *Arabidopsis thaliana* peroxidase (52.49% sequence similarity). Figure 3a illustrates the generated 3D structures of *Aspergillus niger* AN512 peroxidase. Furthermore, the I-TASSER web server was also used to generate high-quality model predictions of 3D structure (Fig. 3b) and biological function of protein *Aspergillus niger* AN512 peroxidase protein.

### Validation of homology model

**Fig. 4:**
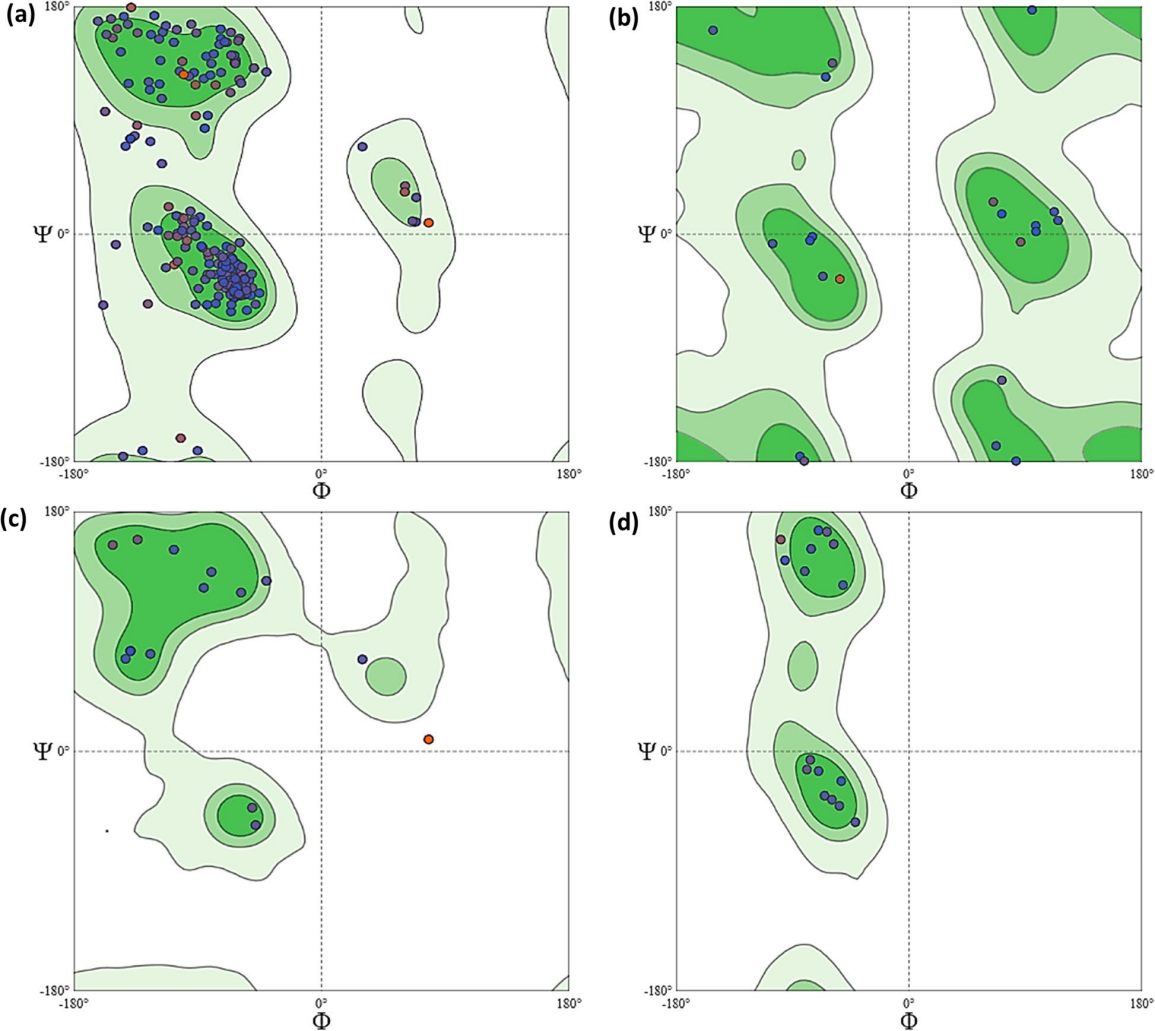
Ramachandran’s plot calculations on the 3D models of peroxidase of *Aspergillus niger* AN512 computed by the SWISS-MODEL web-server to show the favored regions for backbone dihedral angles against of amino acid residues in protein structure **a**) General (No Proline or Glycine) **b**) Glycine Only **c**) Pre-Proline Only **d**) Proline only

To evaluate the predicted 3D structure of the homology model, Ramachandran’s plot of the model was constructed to determine the stereochemical quality of the protein structure by analyzing residue-by-residue geometry. The backbone conformation and overall stereochemical quality of cellulase of *Aspergillus niger* AN512 were calculated by analyzing the phi (Φ) and psi (ψ) torsion angles, and the results were illustrated in the Ramachandran plots in Fig. 4.

### Determination of binding site

**Fig. 5:**
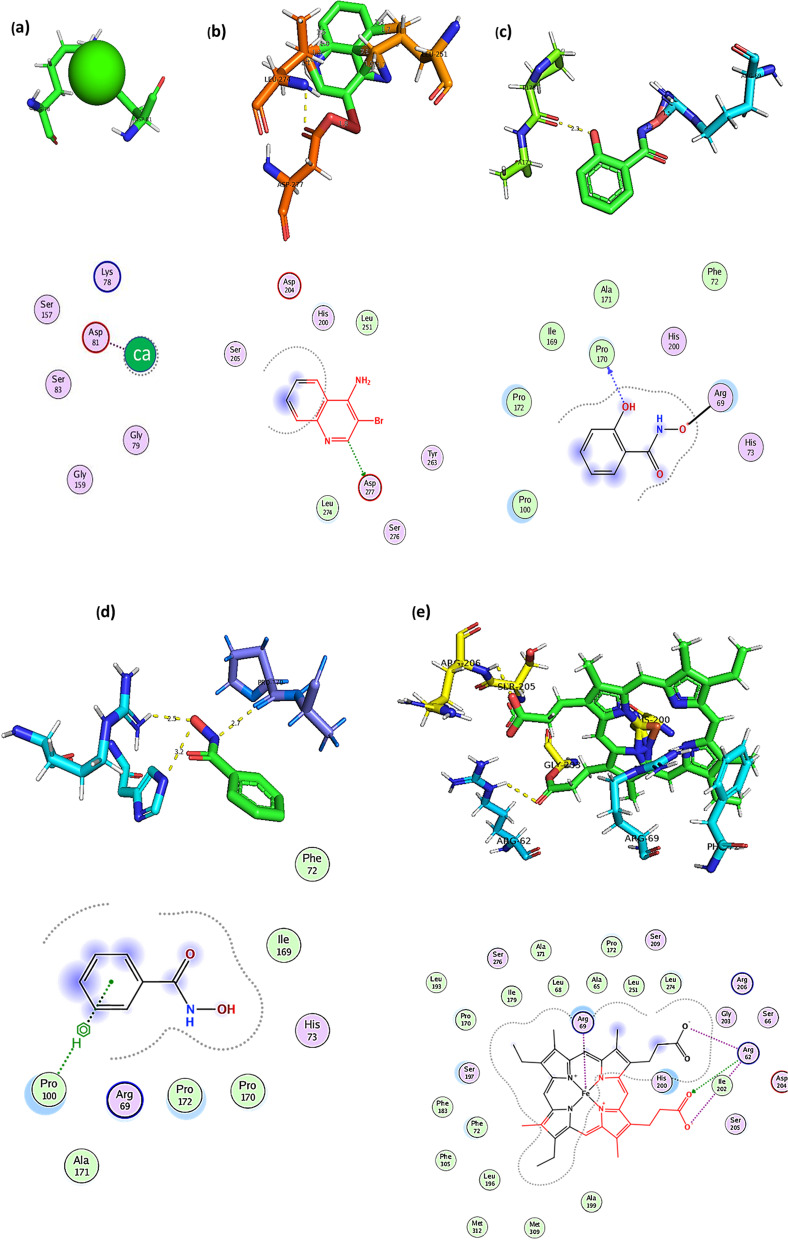
Predicted binding sites in complex with ligands

Biological annotations of the target protein were measured by COACH and COFACTOR based on the I-TASSER structure prediction. While COFACTOR uses structure comparison and protein-protein networks to deduce protein functions (ligand-binding sites, EC, and GO), COACH is a meta-server technique that collects various function annotation results (on ligand-binding sites) from the COFACTOR, TM-SITE, and S-SITE programs. According to a prediction by I-TASSER algorithm for the protein 3D structure, 5 ligands, calcium (2+) at binding site residues (74, 77, 78, 79, 81, 83), b) 3-bromoquinolin-4-amine (2NW) at binding site residues (200, 202, 203, 204, 205, 266, 273, 274, 277), N,2-dihydroxybenzamide (SHA) at binding site residues (69, 72, 73, 100, 170) were detected Fig. 5.

## Discussion

The concentration of CaCl_2_ solution is an important factor affecting the transformation efficiency of competent cells [25]. Li et al. reported that the transformation efficiency of competent cells increased by rising the concentration of CaCl_2_ solution an reached its maximum at 75 mM and then decreased rapidly when the concentration exceeded 100 mM [26]. This result is slightly similar to result in the present study as maximum transformation efficiency reached 50 mM and decreased as CaCl_2_ concentration increased until 150 mM. This inverse proportion between CaCl_2_ concentration and transformation efficiency may be due to lipid array on cell membrane is destroyed by 75 to 100 mM Ca^2+^, and then, a liquid crystal would be formed. The bacteria would be swollen when incubated in hypotonic calcium chloride solution, at 0 °C, and DNA in the mixture can be formed into hydroxyapatite (anti-DNase) and then stick to the surface of cells [27, 28]. The cellular absorption ability of exogenous DNA will be increased after heat shock at 42 °C. This process may be inhibited, and hence, the transformation efficiency will be decreased when the concentration of calcium chloride solution exceeds 100 mM.

The early logarithmic growth period of *E. coli* was the very important factor, the best growth condition, and easily be induced when the OD_600_ meets 0.35 to 0.45 Meanwhile, during this phase, the bacteria are in the best tolerance condition suffering the physical damage, and this could increase the transformation efficiency [29, 30]. Sambrook and Russell [16] found out that the absorptivity of *E. coli* obviously decreased because of the mutation of *hofQ* during the stationary phases.

Chan et al. [25] reported that centrifugation time had little effect on transformation efficiency, but when the time is exceeding 10 min, some dead bacteria would form sediment together with activity bacteria, which could degrade transformation efficiency to a certain extent. On the other hand, almost all of active bacteria could form sediment when centrifugation time was 5 min. So, using centrifugation time from 5 to 10 min for preparation of competent cells will give maximum efficiency.

Catalase-peroxidase gene from the pathogenic fungus *Penicillium marneffei* was isolated by Pongpom et al. [31], and DNA sequence analysis of this gene revealed an ORF encoding a 748 amino acid polypeptide with a predicted molecular mass of 82.4 KDa within the amino acid sequence was 45/69% identical to that of catalase-peroxidases from many bacteria and fungi [29, 31].

The *E. coli* cloned strain was similar to that reported by Falade et al. [1] They isolated freshwater bacterial strains capable of peroxidase production by applying the same identification technique. Also, Al-Senaidy and Ismael [32] obtained peroxides enzyme with 55 KDa. Earlier reports also performed on bacterial peroxidases purified from *Bacillus* sp. VUS Dawkar et al. [33] showed peroxides enzyme with molecular weight of 43 KDa. Isolation and cloning of fungus per-K gene in *E. coli* expression host have numerous advantages including easy extraction method and huge productivity in shorter time in comparison with fungus isolate. Also the used *E. coli* host is more safe than *Aspergillus niger* isolate on the human health in case of using it for commercial production of the enzyme.

Ammonium sulfate is a salt used in the precipitation of enzymes due to its high solubility; therefore, it was used in the precipitation of different enzymes [34]. Partial purification was performed by gradual saturation ratios ranged from 40 to 90% of ammonium sulfate to precipitate crude enzyme, and then, the peroxidase activity was checked in each fraction to check the most suitable saturation according to precipitation of peroxidase enzyme. A total of 90% of ammonium sulfate was given completely precipitation of the enzyme with maximum enzyme activity which is similar to these reported by Kalyani et al. [35] and Mustafa [36]. The result of peroxidase activity was in approval with the studies obtained by Falade et al. [1] who isolated two bacterial strains capable for producing a significantly high peroxidase activity with 5250 and 5833 U/L for both strains. Other studies reported a lower [24] peroxidase activity for different *Streptomyces* strains with 270 and 535 U/L. [37, 38] The reason for different peroxidase production between different isolates is not yet clearly understood; however, the excessive production of peroxidase in the cloned *E. coli* strain confirmed the successful recombinant process with the desired results obtained. Peroxidase-producing microbes possess high potential for industrial applications. One of the main significantly important application is dye treatment process in wastewater streams. Peroxidase-producing microorganisms also serve as important cause in the pretreatment of lignocellulose material which eventually leads to the conversion of lignocellulose, complex molecules to ethanol. Fungi contain diverse important genes encoded several proteins and enzymes used in medical, pharmaceutical, and industrial uses [2, 39–41]. The physical parameters of the obtained protein were predicted, and results showed that *Aspergillus niger* AN512 peroxidase has a calculated isoelectric point pI value of 6.98 and a higher thermal stability. The amino acid sequences of *Aspergillus niger* AN512 peroxidase were subjected to homology modeling via SWISS-MODEL web server to generate the 3D structures of the peroxidase enzyme. The Ramachandran’s plots of the model were constructed to determine the stereochemical quality of the protein structure by analyzing residue-by-residue geometry, and results revealed that the obtained model quality is high according to constructed Ramachandran’s plots.

## Conclusion

This study showed that the optimum conditions for a successful transformation of the PER-K gene of *Aspergillus niger* AN 512 inside *E. coli* BL21-T7 were include 50 mM CaCl_2_ concentration, 20-min centrifugation time, 200 rpm shaking speed, and growth optical density of 0.45. PCR detection of plasmid DNA was through amplification of UA3, AmpR, and PER-K genes. Partial sequencing of the PER-K gene (under submission in NCBI GeneBank) has an activity of 3924 U/L and molecular weight of 55 KDa. Recombinant peroxidase enzyme technique can be used to achieve large-scale production for this enzyme for feasible and efficient industrial applications.

## Supplementary Information

**Additional file 1: Fig. S1.** Yeast episomal vector with a UA3 marker for construction of lacZ fusions. The MCS is reversed in YEp356. **Fig. S2.** DNA sequences and Agarose gel electrophoresis analysis of PCR product of Per-K, UA3 and AmpR amplicons. **Fig. S3.** Phylogenetic tree of Per-K gene sequence of this study and the most genetically related taxa on NCBI, this tree was designed through MEGA 7 software. **Fig. S4.** DNA nucleotides alignment between our Per-K query and the most related two DNA sequences, alignment run through online Clustal Omega and finished through Jalview software. **Fig. S5.** Complete gene sequence open reading frame (ORF) started from 141 to 1145 bps. **Table S1.** Restriction endonucleases used in fungus genomic DNA and vector digestion. **Table S2.** Digestion buffers.

## Data Availability

All authors declare that the data supporting the findings of this study are available within the article.
