# Supplementary material for: Molecular docking and nucleotide sequencing of successive expressed recombinant fungal peroxidase gene in E.coli
Source: J Genet Eng Biotechnol. 2022 Jul 1;20:94. doi: 10.1186/s43141-022-00377-6 (PMC9249955; doi:10.1186/s43141-022-00377-6)
Supplement: Supplementary file 1 — Additional file 1: Fig. S1. Yeast episomal vector with a UA3 marker for construction of lacZ fusions. The MCS is reversed in YEp356. Fig. S2. DNA sequences and Agarose gel electrophoresis analysis of PCR product of Per-K, UA3 and AmpR amplicons. Fig. S3. Phylogenetic tree of Per-K gene sequence of this study and the most genetically related taxa on NCBI, this tree was designed through MEGA 7 software. Fig. S4. DNA nucleotides alignment between our Per-K query and the most related two DNA sequences, alignment run through online Clustal Omega and finished through Jalview software. Fig. S5. Complete gene sequence open reading frame (ORF) started from 141 to 1145 bps. Table S1. Restriction endonucleases used in fungus genomic DNA and vector digestion. Table S2. Digestion buffers. [file 43141_2022_377_MOESM1_ESM.docx]

**Fig. S1**: Yeast episomal vector with a UA3 marker for construction of lacZ fusions. The MCS is reversed in YEp356.

**Fig. S2:** DNA sequences and Agarose gel electrophoresis analysis of PCR product of Per-K, UA3 and AmpR amplicons.

**Fig. S3:** Phylogenetic tree of Per-K gene sequence of this study and the most genetically related taxa on NCBI, this tree was designed through MEGA 7 software.

**Fig. S4:** DNA nucleotides alignment between our Per-K query and the most related two DNA sequences, alignment run through online Clustal Omega and finished through Jalview software.

**Fig. S5:** Complete gene sequence open reading frame (ORF) started from 141 to 1145 bps**.**

**Fig. S1**.


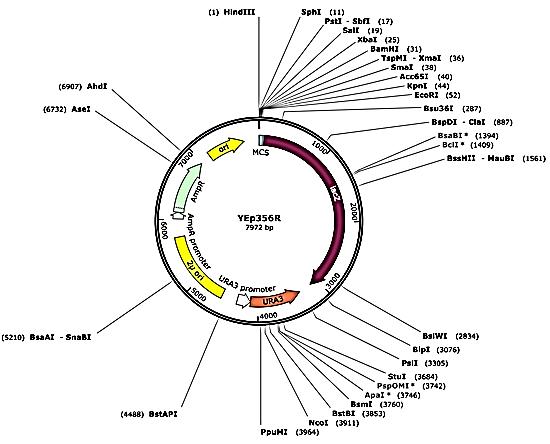


**Fig. S2:**

| DNA sequence of Per-K amplicon  CCAAATCTCCTCTTCCTTATATCACACAATCACACGAAGAGAGCTAGCCAGCTAAAATGGCTCTTTCTATGAACTTGCTTCTAGTTTTCACTTCTCTTGCTCTTGTTCCTCTATGCTTTGCTAGCTATGGTGGGCCTAGTTCCAGCGGCTTCCTCTACCCTCAGTTTTATGATCACTCTTGCCCAAAAGCACAGGAGATTGTGAAGAGCGT  DNA sequence of UA3 amplicon GCAAGGGCTCCCTAGCTACTGGAGAATATACTAAGGGTACTG TGACATTGCGAAGAGCGACAAAGATTTTGTTATCGGCTTTATTGCTCAAAGAGACATGGGTGGAAGAGATGAAGGTTACGATTGGTTGATTATGACACCCGGTGTGGGTTTAGATGACAAGGGAGACGCATT  DNA sequence of Amp^R^  GAGTATTCAACATTTCCGTGTCGCCCTTATTCCCTTTTTTGCGGCATTTTGCCTTCCTGTTTTTGCTCACCCAGAAACGCTGGTGAAAGTAAAAGATGCTGAAGATCAGTTGGGTGCACGAGTGGGTTACATCGAACTGGATCTCAACAGCGGTAAGATCCTTGAGAGTTTTCGCCCCG |
| --- |

| **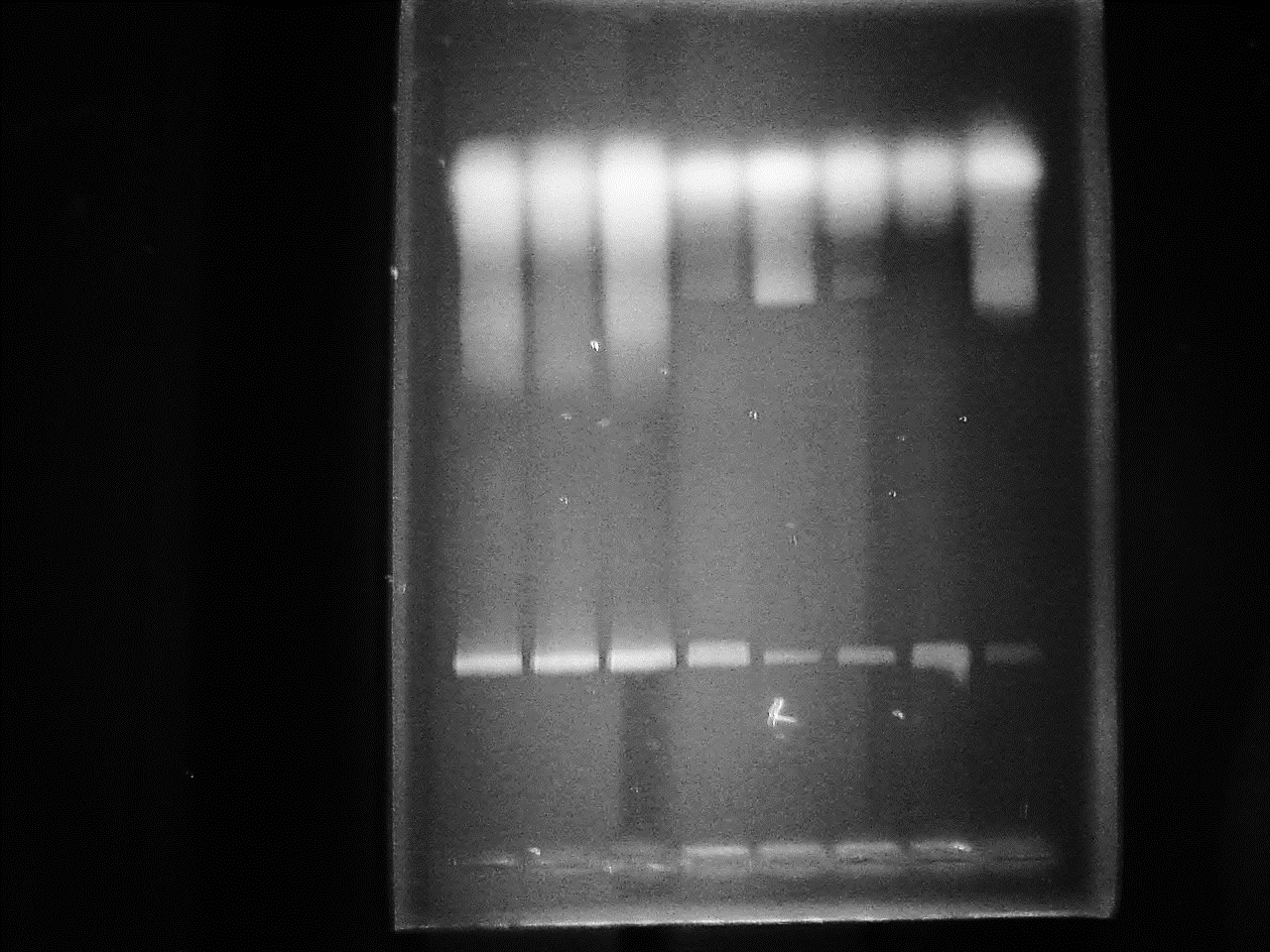**  ***Per-K***  **211** | **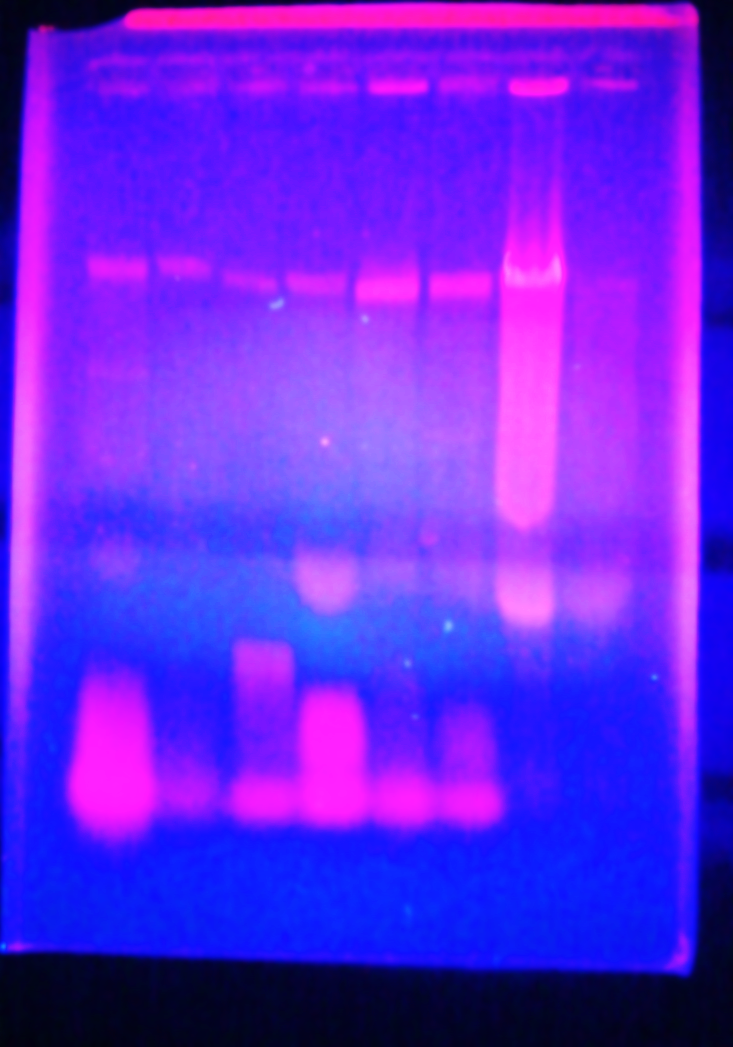**  ***UA3***  **175** | **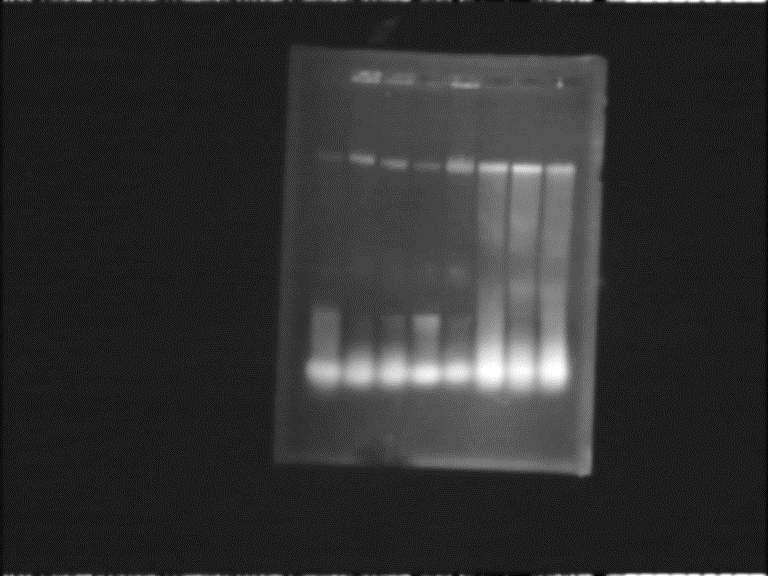**  ***AMP^R^***  **179** |
| --- | --- | --- |

**Fig. S3.**


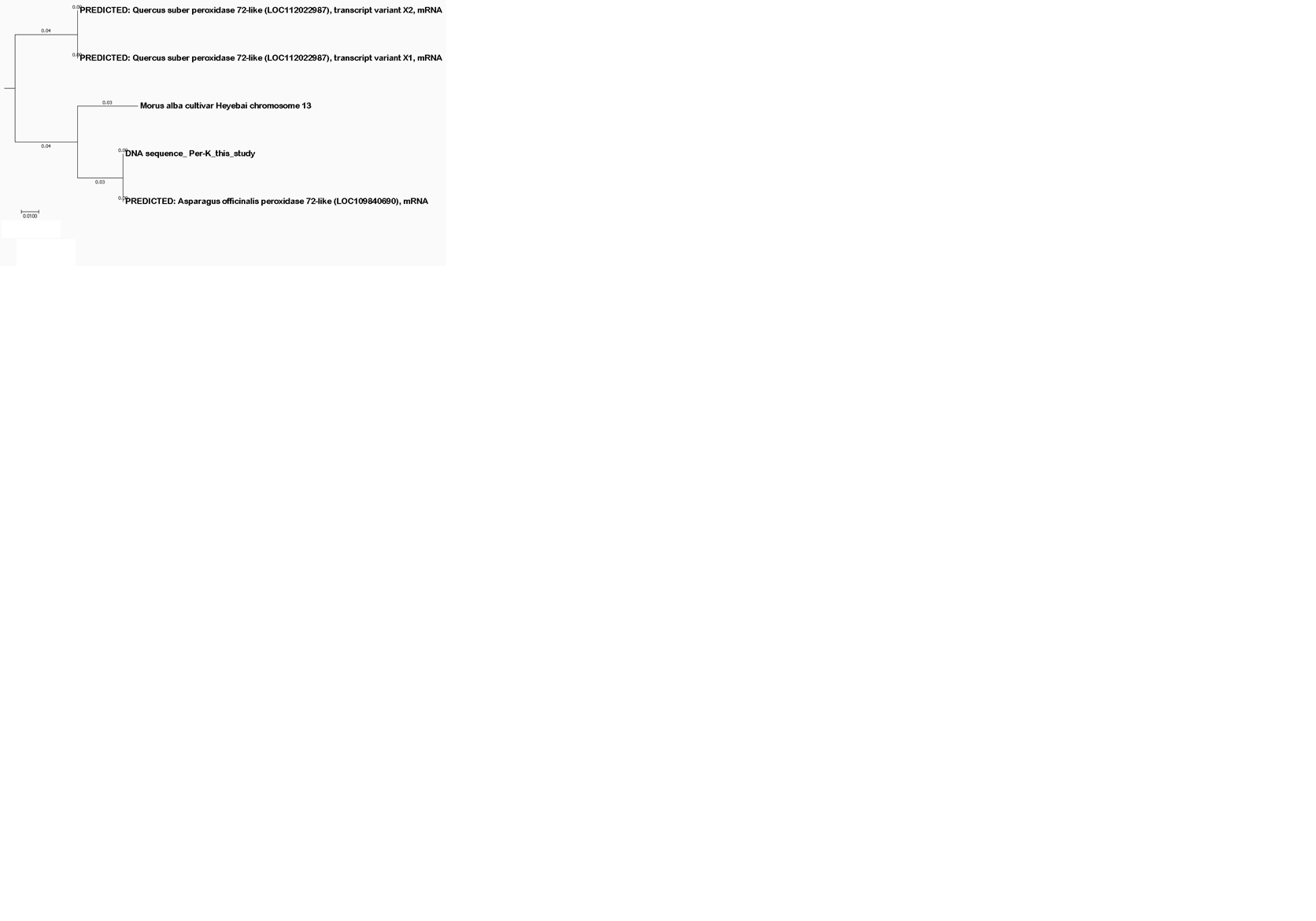


**Fig. S4.**


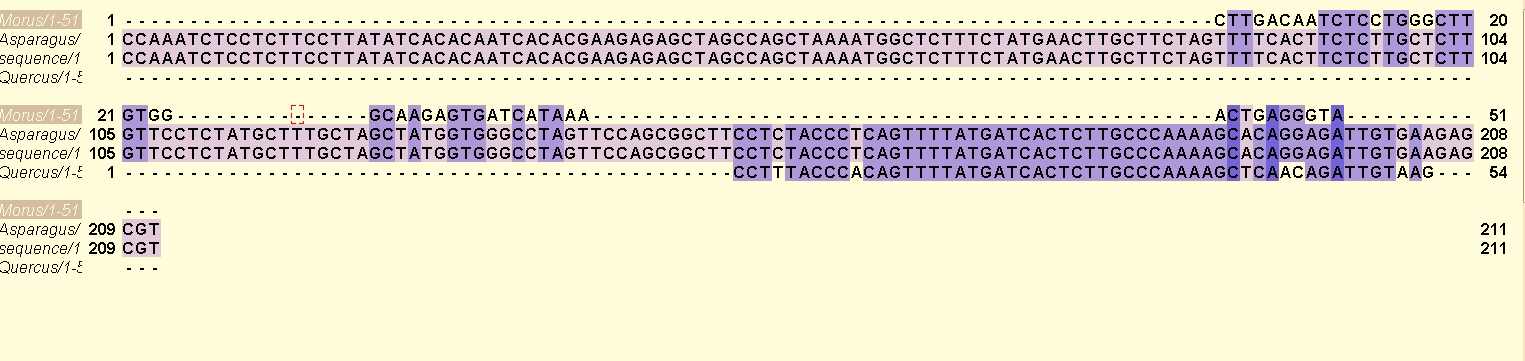


**Fig. S5**


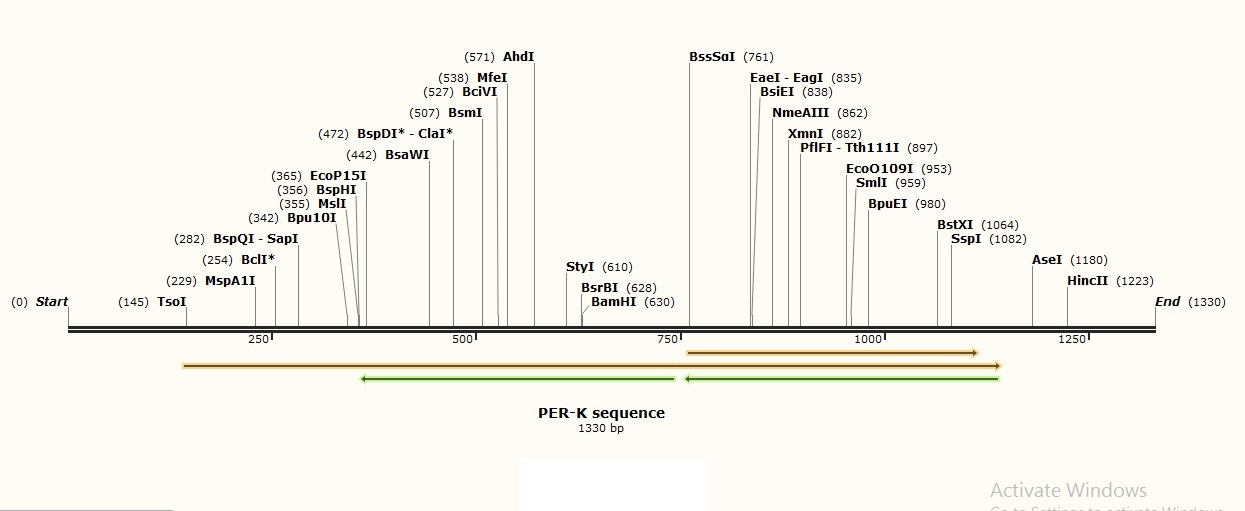


**Table (S1):** Restriction endonucleases used in fungus genomic DNA and vector digestion

| Restriction enzyme | Usage |
| --- | --- |
| *Hin*dIII (1) | Isolation desired gene by digestion of Fungus DNA, also used to open vector at site 1 sticky ends in MCS |
| *Sa*lI (2) | Isolation desired gene by digestion of Fungus DNA, also used to open vector at site 19 sticky ends in MCS |

**Table (S2):** Digestion buffers

| Buffer | Concentration |
| --- | --- |
| Restriction enzyme buffer for SalI digestion  1X Buffer O (for 100%) 50 mM Tris-HCl (pH 7.5), 10 mM MgCl 2, 100 mM NaCl, 0.1 mg/mL BSA. | nuclease-free water 15 µL |
|  | 10X Buffer O 2 µL |
|  | DNA (1 µg/µL) 2µL |
|  | SalI 2 µL |
| Restriction enzyme buffer for HindIII digestion  1X Buffer R (for 100%) 10 mM Tris-HCl (pH 8.5), 10 mM MgCl2,100 mM KCl, 0.1 mg/mL BSA. | DNA (1 µg/µL) 2 µL |
|  | HindIII 2 µL |
|  | 10X Buffer R 2 µL |
|  | nuclease-free water 15 µL |
| incubate in water bath at 37^°^C for 3-7h | |
